# Context-Related Acoustic Variation in Male Fallow Deer (*Dama dama*) Groans

**DOI:** 10.1371/journal.pone.0021066

**Published:** 2011-06-13

**Authors:** Benjamin D. Charlton, David Reby

**Affiliations:** 1 Department of Cognitive Biology, University of Vienna, Vienna, Austria; 2 School of Psychology, University of Sussex, Sussex, United Kingdom; Texas A&M University, United States of America

## Abstract

While social and behavioural contexts are known to affect the acoustic structure of vocal signals in several mammal species, few studies have investigated context-related acoustic variation during inter-sexual advertisement and/or intra-sexual competition. Here we recorded male fallow deer groans during the breeding season and investigated how key acoustic parameters (fundamental frequency and formant frequencies) vary as a function of the social context in which they are produced. We found that in the presence of females, male fallow deer produced groans with higher mean fundamental frequency when vocal males were also present than they did when no vocal males were in close vicinity. We attribute this to the increased arousal state typically associated with this context. In addition, groan minimum formant frequency spacing was slightly, but significantly lower (indicating marginally more extended vocal tracts) when males were alone than when potential mates and/or competitors were nearby. This indicates that, contrary to our predictions, male fallow deer do not exaggerate the acoustic impression of their body size by further lowering their formant frequencies in the presence of potential mating partners and competitors. Furthermore, since the magnitude of the variation in groan minimum formant frequency spacing remains small compared to documented inter-individual differences, our findings are consistent with the hypothesis that formants are reliable static cues to body size during intra- and inter-sexual advertisement that do not concurrently encode dynamic motivation-related information.

## Introduction

Recent studies of mammal communication have highlighted the multi-component nature of vocal signals (for review see [1]), and shown how the inter and intra-individual variation of the spectral components that compose these signals can simultaneously convey information on both static (long-term) and dynamic (short-term) attributes of callers. Vocalisations have been found to encode information on static attributes of callers such as their sex [2], [3], body size [3]–[9] or identity [10]–[15], as well as information on dynamic attributes of callers such as their emotional or motivational state [10], [16], [17]. Dynamic attributes are typically affected by a combination of internal factors such as the physiological state of the caller [18], [19] and external factors such as the social contexts of emission [20]–[28]. While several detailed investigations of the acoustic structure of mammal signals have identified the simultaneous presence of static and dynamic variation, there is an obvious potential conflict between the two types of information: components that encode information on static attributes should be characterised by limited variability, while components which encode information on dynamic attributes should be free to co-vary with the traits they express [29].

In the contexts of agonistic and sexual interactions, the “size” or “frequency” code hypothesis offers an evolutionary scenario whereby specific features of vocal signals known to encode honest information on static attributes of callers, such as their size, can simultaneously encode short-term, dynamic, motivational information [30]. This theory predicts that callers may evolve the ability to make ritualised use of relatively small variations of otherwise constrained parameters during social interactions. For example, in a species where spectral components correlate negatively with body size, callers may lower these components by a small amount in order to exaggerate the acoustic expression of their body size in order to sound more aggressive, or raise these components in order to minimise the acoustic expression of their body size and therefore sound more submissive [30]. However, the possible ritualised use of acoustic cues to static attributes has not been investigated in the context of nonhuman mammal vocal interactions.

The sexually-selected vocal displays of male fallow deer (*Dama dama*) constitute a useful model for investigating short-term variation of vocal behaviour. Early studies of vocal behaviour in fallow bucks focused on the function of calling rates: males who initiated vocal activity and remained vocal on most days during the rut achieved higher numbers of copulations [31] and average groaning rates were found to be related to mating success [32]. Investigations of short-term variation in calling rates found that males groaned at higher rates when females were present, and that males with females increased their groaning rates when vocal males were in close vicinity [33]. More recent studies of groaning in fallow deer have extensively described inter-individual variation in the acoustic structure of groans [12] as well as medium-term (during the rut) and long-term (lifespan) intra-individual variation [29], [34].

The acoustic structure of the fallow deer groan is best described in terms of its source- (fundamental frequency) and filter- (formant frequency) related components [6], [12], [29], [34], [35]. Male fallow deer groans are short, stereotypical vocalisations with an extremely low and sexually dimorphic fundamental frequency (F0) [11], [35], [36]. Although the basis for the inter-individual F0 variation in fallow deer groans remains unknown, in humans a lower F0 is associated with higher androgen levels [37], and correlational investigations have shown that higher-ranking, more dominant males produce groans with lower minimum F0 [6]. In addition, vocal tract resonances (hereafter termed formants) are reliable cues to body size in male fallow deer groans [6], as they are in the calls of several other male mammals [3]–[5], [7]–[9].

Here, we recorded free-ranging male fallow deer during the breeding season in order to determine whether variation in social context affects the acoustic structure of fallow deer ‘common groans’. More specifically, following McElligott & Hayden's [33] investigation of the effect of context on short-term rates of groaning, we investigated the effect of key proximate social factors on the acoustic structure of male fallow deer groans: the presence or absence of female(s) and vocal male(s). Along the “frequency code” theory [30], and previous findings in red deer (*Cervus elaphus*) [38], [39] we predict that fallow bucks should maximally extend their vocal tracts and therefore fully lower their formant frequencies when other vocal males are nearby and/or when females are in close proximity, as a means of maximising their apparent body size to competitors and potential mates. We have no strong *a priori* prediction for the affect of social context on groan F0. However, since lower minimum F0 is an established indicator of dominance in fallow bucks [6], we may expect mean F0 to be lower when males are in close proximity.

## Results

### Groan mean F0

**Figure 1:**
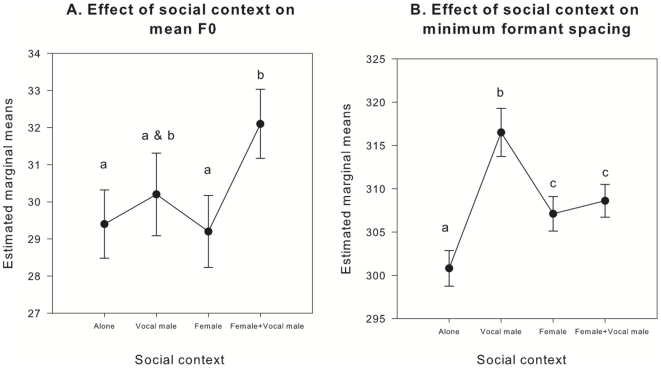
Estimated marginal means ± SE for the effect of social context (alone, with a vocal male, with a female, with a female and a vocal male) on mean F0 (A) and minimum ΔF (B). Mean responses sharing the same letter are not significantly different.

The social context of recording had a significant effect on groan F0 (F_3, 246_ = 3.637, p = 0.013). The pair-wise comparisons showed that males with females produced groans with higher mean F0 when another vocal male was present (mean ± S.E. = 32.1±0.93 Hz) than they did when no other vocal male was close by (mean ± S.E. = 29.2±0.97 Hz; P = 0.032) or when they were alone (mean ± S.E. = 29.4±0.92 Hz; P = 0.030), but not significantly higher than when only a vocal male was present (mean ± S.E. = 30.2±1.11 Hz; P = 0.359) (see Figure 1A). No significant differences were detected in the mean F0 of male groans between the other social contexts (see Figure 1A).

### Groan minimum ΔF

The social context also had a significant effect on groan minimum ΔF (F_3, 238_ = 8.640, p<0.001): groan minimum ΔF was lower (indicating more fully extended vocal tracts) when males were alone (mean ± S.E. = 300.8±2.05 Hz) than it was when vocal males (mean ± S.E. = 316.5±2.78 Hz; P<0.001), females (mean ± S.E. = 307.1±1.99 Hz; P = 0.041) or vocal males and females (mean ± S.E. = 308.6±1.89 Hz; P = 0.003) were in close attendance (see Figure 1B). In addition, males produced groans with higher minimum ΔF when they were with other vocal males (mean ± S.E. = 316.5±2.78 Hz) than they did in any other context (see Figure 1B).

## Discussion

The results of this study indicate that the presence of mating partners and competitors has a significant effect on the vocal behaviour of male fallow deer, with frequency components of male groans varying between different social contexts. Specifically, we have shown that the mean F0 of male fallow deer groans increases when males are with females and other vocal males are in close proximity. In addition, fallow bucks that were alone produced groans with lower minimum ΔF than they did when vocal males and/or females were in close attendance, and higher minimum ΔF when vocal males were in close vicinity than they did in any other social context.

The higher mean F0 of groans produced by males with females when other vocal males were nearby could plausibly reflect greater arousal levels around potentially dangerous rivals. Indeed, vocal males are considered to be actively rutting [40] and, therefore, likely to constitute a threat. Moreover, previous work on this species showed that the rate of male groans increased when males were with females and other vocal males were close by [33], and higher rates of vocal activity are likely to signal increased motivation [41]. In addition, work on nonhuman mammals has shown that increased mean F0 is associated with increased arousal [10] and, from a vocal production perspective, high arousal levels could lead to increased sub-glottal pressure and dynamic strain on the vocal folds, leading to higher mean F0 [42]. Interestingly, the mean F0 of male fallow deer groans did not appear to change according to whether a female was present or not (see Figure 1), consistent with previous studies suggesting that dynamic variation in groaning behaviour is primarily directed at male competitors [33].

Contrary to our predictions, we found that bucks extended their vocal tracts less fully in the presence of potential mating partners and competitors than they did in the absence of both and thus, do not appear to make ritualistic use of size-related formant information to maximise the acoustic impression of their body size in these contexts. An explanation for this could be the very high groaning rates that male fallow deer are known to produce when there are females or vocal males nearby [circa 55 groans per minute: 33], which may make it more difficult for them to always lower the larynx to the maximum [unlike in the closely related red deer which only roars up to 8 times per minute: [43]]. However, the total magnitude of the observed variation (∼5%) remains small compared to the natural variation in the minimum formant spacing of adult male fallow deer groans of around 16% [minimum = 281 Hz, maximum = 326 Hz: 12] and, therefore, may not reflect a meaningful difference between a large, medium, and small male representative of the population i.e. males would probably still be categorised as small, medium or large by receivers.

It is also important to note that we cannot automatically assume that all the context-related differences in minimum ΔF and F0 that we report here are perceptible by fallow deer. Studies of human voice perception show that while listeners can discriminate differences in F0 as low as 2%, this rises to about 9% when F0 approaches 40 Hz [44], below which the mean F0 of most fallow deer groans falls [12], [34]. Similarly, human listeners cannot perceive shifts in formant spacing or apparent vocal tract length of less than 4% [44], [45]. While we cannot generalise human perceptual abilities to fallow deer, whether the variation reported above is perceptually relevant for this species remains to be demonstrated. We suggest that future studies investigate the perceptual abilities of male and female fallow deer, using habituation–discrimination tests and re-synthesised groan stimuli with different levels of F0 and formant spacing manipulation [46].

In conclusion, the results of the current study show that the mean F0 of male fallow deer groans increases when other vocal males are in close proximity, and do not support the hypothesis that males maximise the acoustic impression of their body size during close-range intra-sexual assessment and inter-sexual contexts. While the observed variation in F0 probably reflects variation in arousal, the higher minimum ΔF of groans produced by males when other vocal males are nearby may reflect a trade off between groaning rate and laryngeal lowering in these contexts. Nevertheless, the observed variation in minimum ΔF remains small compared to inter-individual variation reported for this species, and its perceptual and functional significance remain to be investigated. Furthermore, our findings are consistent with the hypothesis that formants are mostly static cues to body size during intra- and inter-sexual advertisement [6] that do not concurrently encode dynamic motivation-related information. Indeed, recent work on red deer has shown that formants are likely to function as static indices of body size in female mate choice contexts [39], [47] and when harem holders assess rivals [38]. Accordingly, we suggest that future studies on fallow deer use resynthesis techniques and playback experiments to further investigate the relative importance of size-related formant information during intra-sexual assessment and female mate choice contexts.

## Materials and Methods

### Ethical statement

This work follows the Association for the study of Animal Behaviour/Animal Behaviour Society guidelines for the use of animals in research, and was approved by the Ethics Committee of the Department of Psychology at the University of Sussex.

### Study site and animals

A free-ranging population of fallow deer were observed during the 2004 breeding season (7th to 26th October) at Petworth Park, West Sussex, in the U.K. During the study period there were approximately 1000 deer in the park. Because the males in our study were not individually marked, unique antler shape and coat markings were used to identify individuals from multiple digital photographs taken at the time of each recording using a Nikon Coolpix 8700 (8 megapixel, 10× optical zoom). Only recordings from individuals that could be identified with absolute certainty were retained in the analysis.

### Recordings and contextual settings

**Figure 2:**
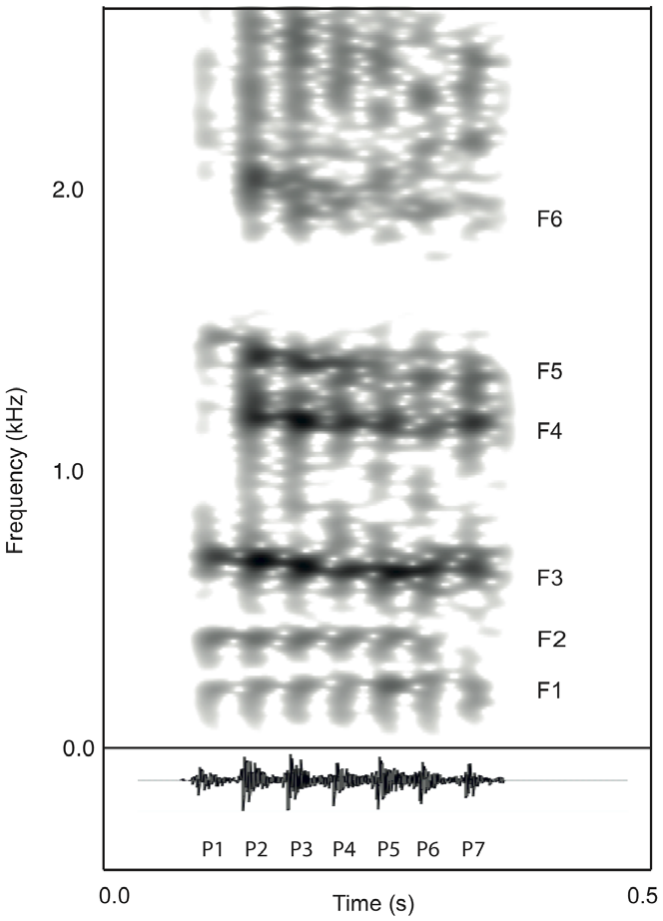
Spectrogram (A) and waveform (B) of a male fallow deer groan. Male fallow deer groans are low-pitched vocalisations characterised by a rapid downward shift in formant frequencies across the call (spectrogram settings: FFT method, window length = 0.05 s, time step = 0.01 s, Gaussian window shape, dynamic range = 30 dB).

Groans were recorded from each of 18 male fallow deer at distances of 10–30 metres using a Marantz PMD 670 CF-card recorder and a Sennheiser MKH 416 P48 directional microphone (sampling rate of 44.1 kHz, 16 bits amplitude resolution). The sound files were transferred to an Apple Macintosh Macbook computer for visual inspection using narrow band spectrograms (see Figure 2: FFT method, window length = 0.03 s, time steps = 250, frequency steps = 1000, Gaussian window shape, dynamic range = 50 dB) and recordings with high levels of background noise were discarded. In total we used 269 groans from 11 males recorded on nine separate days. For each of the recording sessions we noted whether a vocally active male was within approximately 30 metres of the focal male. Vocal males are likely to constitute a greater threat because they are actively rutting [40]. We also noted whether any females were within 30 metres of the focal male (following [33]), and if the focal male was a harem holder.

### Acoustic analysis

We used Praat DSP package version 5.0.29 (www.praat.org) for the acoustic analysis. To measure F0 parameters we extracted the F0 contour of groans using the ‘To pitch (ac) command’ (time step = 0.01 s; minimum and maximum F0 = 20 Hz and 50 Hz, respectively). Time-varying numerical representations of the F0 contour were compared with the F0 contour as visualized on a spectrogram and checked to see if the autocorrelation algorithm was correctly tracking the F0. In cases where a harmonic or a sub-harmonic were tracked instead of the fundamental frequency (octave jumps), numerical representations of the F0 contour were manually adjusted using the ‘Edit’ window in Praat, and the resulting F0 contour was played back as a sine wave for comparison with the original recording.

As stated in the introduction, male fallow deer retract their larynx towards the sternum during vocalisation, resulting in a lowering of formants and formant spacing (ΔF) across the call [35]. Consequently, to quantify the minimum ΔF achieved when the larynx is fully retracted, we took the last pulses of each groan (∼0.05 s in duration) for our analysis. The frequency values of the first six formants were measured using Linear Predictive Coding (LPC; ‘To Formants (Burg)’ command in Praat) and the following analysis parameters: time step: 0.01 s; window analysis: 0.05 s; maximum formant value: 2300 Hz; maximum number of formants: 7; pre-emphasis: 6000 Hz. Since the first two formants are often poorly represented and vocal tract elongation has a minimal effect on them [35], the upper formant values (F3, F4, F5 & F6) were used to estimate ΔF during each groan using the linear regression method described by Reby and McComb [9].

### Statistical analysis

We used linear mixed-effect models (LMM's) fitted with maximum likelihood estimation to examine relationships between acoustic features of male groans and the different contextual settings. Our acoustic measures (mean F0 and minimum ΔF) were entered as dependent variables and the social context was entered as a fixed factor categorical independent variable. Q-Q plots and scatter plots of residuals were inspected to ensure the dependant variables were normally distributed. The different contextual settings were as follows: no vocal adult male(s) or female(s) within 30 metres of the focal male; vocal adult male within 30 metres of the focal male; female(s) within 30 metres of the focal male; female(s) and vocal male(s) within 30 metres of the focal male. Pair-wise comparisons with Bonferroni adjustments were used to examine differences between the different social contexts. In addition, we entered subject identity as a random factor in each of our LMM's to control for repeated measures taken from the same individual. We also included the recording date and if the focal male was a harem holder as covariates, and used a model selection criteria based on the Akaike's Information Criteria (AIC), in which the model having the lowest AIC value is chosen [48]. Non-significant interaction terms were always removed from the model [49]. For the effect of social context on mean F0 the model with the lowest AIC value did not include the recording date or if the focal male was a harem holder as covariates. For groan minimum ΔF, the best model included the recording date, whether the focal male was a harem holder or not, and the interaction terms harem*recording date and social context*recording date. SPSS version 16 for Mac OS 10.5 was used to run the linear mixed effect models, significance levels were set at 0.05, and two-tailed probability values are quoted.
